# Emerging role of lymphatic vessels in reverse cholesterol transport

**DOI:** 10.18632/aging.100570

**Published:** 2013-06-23

**Authors:** Angeli Veronique, Lim Hwee Ying

**Affiliations:** Department of Microbiology, Immunology Programme, Yong Loo Lin School of Medicine, National University of Singapore

As a major constituent of cell membrane, cholesterol is essential for cellular homeostasis through the control of membrane fluidity and permeability. Abnormal levels of cholesterol can have important cellular consequences and may lead to diseases such as atherosclerosis and type II diabetes. Therefore, cells have developed complex mechanisms to regulate the abundance of intracellular cholesterol. Among these mechanisms, the process referred to as reverse cholesterol transport (RCT) has been shown to be critical for the removal of excess cholesterol from peripheral tissues and its transport to the liver via plasma for excretion [1]. RCT is initiated when cells including macrophages efflux out the excess of cholesterol to prevent intracellular over-accumulation. This step is tightly regulated through the action of ATP-binding cassette transporters ABCA1, ABCG1 and scavenger receptor class BI (SR-BI) and high density lipoprotein (HDL) which serves as the major acceptor for cellular cholesterol released in extrahepatic tissues [2]. Following cholesterol efflux, HDL-cholesterol is transported from the tissue back into the blood circulation. In the final step of RCT, the majority of HDL-derived cholesterol is removed from the liver by secretion into the bile [1, 2]. In contrast to the knowledge on the mechanisms by which cholesterol is effluxed out of the cells and its fate after reaching the liver, little information is available on how HDL-cholesterol travels from the peripheral tissues back to the blood circulation.

Since one of the major roles of lymphatic vessels is to drain macromolecules from the interstitial space back to the circulation and lymph is rich in cholesterol and HDL [3], the implication of lymphatics in the transport of lipoproteins from interstitium to blood has been suspected. The recent studies by Martel et al., and Lim et al., now provide convincing evidence for a role of lymphatic vessels in RCT [4, 5]. Indeed, impairment of lymphatic drainage induced by surgical excision in wild-type mice [4, 5] or naturally occurring in mice lacking the expression of one allele of vascular endothelial growth factor-C receptor [4] significantly decreased the efficiency of RCT. Furthermore, Lim et al., showed that the transport of effluxed cholesterol from peripheral tissue to the blood circulation via lymphatics is not passive as anticipated but an active process dependent on HDL and SR-BI [5]. Therefore, these new findings establish that lymphatic vessels tightly regulate RCT through the active transport of HDL-cholesterol.

Accumulation of cholesterol in tissues including arteries and skin is a hallmark of atherosclerosis and xanthomas, respectively, and these features are recapitulated in mouse models lacking apoE (apoE^−/−^) or LDL receptor which develop hypercholesterolemia. Interestingly, we showed previously that skin lymphatic vessels in these hypercholesterolemic mice exhibit structural defects which severely compromise their function [6]. These observations suggest that the accumulation of cholesterol in arteries and skin from hyper-cholesterolemic mice may result from poor lymphatic drainage. We now provide definitive evidence that restoring lymphatic function in apoE^−/−^ mice ameliorates the clearance of cholesterol from skin by supporting RCT [5]. In strong agreement with these findings in skin, Martel et al., showed in an elegant surgical model of aorta transplantation that impeding lymphatic vessel growth interferes with the removal of cholesterol from the transplanted aorta [4]. Altogether, these findings reveal the potential atheroprotective role of lymphatic drainage by actively contributing to RCT.

**Figure 1:**
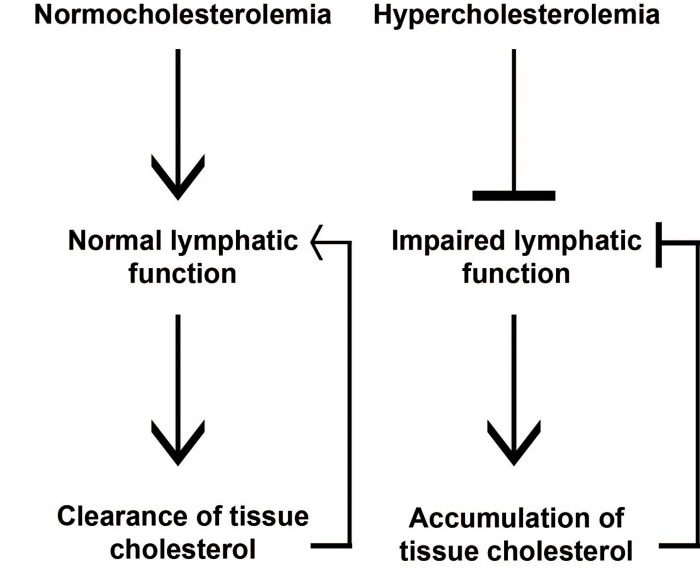
Lymphatic vessels are important for peripheral lipid clearance. Under normocholesterolemic conditions, functional lymphatic vessels regulate cholesterol transport from extra-hepatic tissues by contributing to RCT. Hypercholesterolemic conditions results in dysfunctional lymphatic vessels, which in turn can cause further cholesterol accumulation in tissues.

The relevance of these findings in humans is supported by clinical studies reporting a possible interconnection between lymphatic function and lipid accumulation in tissues (Figure 1). Patients with familial combined hyperlipidemia, a genetic dyslipidemia, which share similar clinical and biochemical features with type II diabetes mellitus, obesity and the metabolic syndrome showed decreased expression of FOXC2 and Prox-1 genes in their adipose tissue [7]. Since these two genes regulate the structure and function of lymphatic vessels these findings support the hypothesis that defective lymphatic drainage may contribute to this lipid disorder. Massive lipid deposits are evident in the edematous tissues of patients with lymphedema and poor lymphatic drainage has been associated with the formation of xanthomas in humans [8]. However, it remains to be determined whether RCT is affected in patients with lymphedema. Interestingly, the use of compression garments to resolve lymphedema ameliorate xanthomatous eruptions in patients [8]. Therefore, promoting efficient lymphatic drainage in lipid-related diseases including atherosclerosis and diabetes whose prevalence will continue to augment as a result of increase in aging population may be an attractive therapeutic intervention that deserves future investigations.
